# Wo kommen wir her? Wo stehen wir? Wo gehen wir hin? Präsidentenrede zur 92. Jahresversammlung der Deutschen Gesellschaft für Hals-Nasen-Ohren-Heilkunde, Kopf- und Hals-Chirurgie, anlässlich des 100. Jahrestages ihrer Gründung

**DOI:** 10.1007/s00106-021-01136-8

**Published:** 2022-03-22

**Authors:** Stefan K. Plontke

**Affiliations:** Universitätsklinik und Poliklinik für Hals-Nasen-Ohren-Heilkunde, Kopf- und Hals-Chirurgie, Martin‑Luther-Universität Halle-Wittenberg, Ernst-Grube-Str. 40, 06120 Halle (Saale), Deutschland


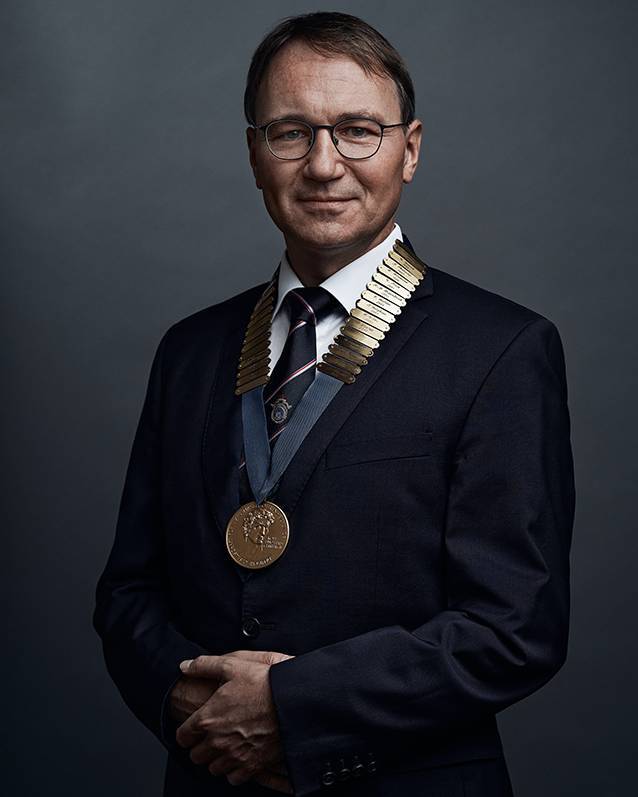


Lieber Mit-Kongresspräsident, lieber Andreas,

liebe Ehrenpräsidenten, lieber Hans-Peter, lieber Herr Maier,

liebe Mitglieder des Präsidiums,

liebe Ellen Lundershausen, Vizepräsidentin der BÄK (Bundesärztekammer),

lieber Dirk Heinrich, Vorsitzender des BV HNO (Deutscher Berufsverband der Hals-Nasen-Ohrenärzte e.V.),

Spectabilis, lieber Herr Gekle,

sehr geehrter Herr Prof. Meller,

liebe Kolleginnen und Kollegen an den Bildschirmen zu Hause und wo auch immer Sie sich eingewählt haben,

in diesem Monat feiert die Deutsche Gesellschaft für Hals-Nasen-Ohren-Heilkunde, Kopf- und Hals-Chirurgie (DGHNO-KHC) ein besonderes Jubiläum. Daher möchte ich mich im Rahmen der Präsidentenrede den Fragen ***„Wo kommen wir her? Wo stehen wir? Wo gehen wir hin?“***, die auch gleichzeitig ein Kongress-Motto darstellen, widmen.

Meinen Ausführungen voranstellen möchte ich einen „Disclaimer“. Der US-amerikanische Philosoph und Schriftsteller Ralph Waldo Emerson (1803–1882) merkte bereits an, dass wir uns dessen bewusst sein sollten, dass unsere besten Ideen meist von anderen stammen („*Our best thoughts come from others.*“).

Und so beginnt es bereits mit dem Titel, denn dieser ist gewählt in Anlehnung an das Vorwort des Hauptwerkes* Das Prinzip Hoffnung* des Philosophen Ernst Bloch (1885–1977), welches er im Exil in den USA schrieb und welches mit den Worten beginnt: „Wer sind wir? Wo kommen wir her? Wohin gehen wir? Was erwarten wir? Was erwartet uns?“ [4]. Ernst Bloch gilt als neomarxistischer Philosoph – und ehe hier Fragen aufkommen: Ich bin kein Marxist. Die Geschichte von Ernst Bloch ist interessant, da er aus den USA in die DDR auf den Lehrstuhl für Philosophie an der damaligen Karl-Marx-Universität Leipzig berufen wurde. Weil er jedoch seine humanistischen Freiheitsideen, z. B. im Zusammenhang mit dem ungarischen Volksaufstand und den Ereignissen des 17. Juni 1953, lehrte, wurde er 1957 aus politischen Gründen von der Universität „emeritiert“ und emigrierte danach in die Bundesrepublik und dort nach Tübingen.

Es gibt viele Zitate, die darstellen, warum es wichtig ist, sich mit der Vergangenheit zu beschäftigen. George Santayana (1863–1952) schrieb 1905 in *The Life of Reason*: „Wer sich seiner Vergangenheit nicht erinnert, ist verurteilt, sie zu wiederholen“ [38].

Und Professor Volker Ladenthin aus Bonn schrieb in der aktuellen *Forschung und Lehre*: „Wir sind eine prassende Moderne, wenn wir glauben, alles ließe sich von jeder Generation immer wieder neu denken“ [25].

Beginnen wir also zunächst mit der Frage:

## „Wo kommen wir her?“

Die Geschichte der HNO-Heilkunde kann grob in vier Phasen eingeteilt werden (Tab. 1).

**Table 1:** 

1.	Die Akkumulation und der Fortschritt gestreuten Wissens bis zur Mitte des 19. Jahrhunderts
2.	Die Gründung der ersten Subspezialitäten Otologie, Laryngologie und Rhinologie ab der Mitte des 19. Jahrhunderts, einschließlich deren Akademisierung: erste Vorlesungen, Spezialsprechstunden und Polikliniken, Krankenhäuser, außerordentliche und später ordentliche Professuren, Zeitschriften, Bücher, Fachgesellschaften, Fachkongresse, Schlüsselerfindungen u. a.
3.	Die Gründung der Otorhinolaryngologie als vereinigtes Fachgebiet Ende des 19. und Anfang des 20. Jahrhunderts
4.	Die Konsolidierung und Weiterentwicklung der Otorhinolaryngologie mit der entsprechenden Anerkennung des Fachgebietes und seiner Einreihung in den obligaten Fächerkanon der universitären Lehre, bahnbrechende Entwicklungen mit – aus der Sicht Deutschlands – Einfluss auf und Export in die Nachbarfächer im 20. Jahrhundert sowie die zunehmende Internationalisierung

**Figure 1:**
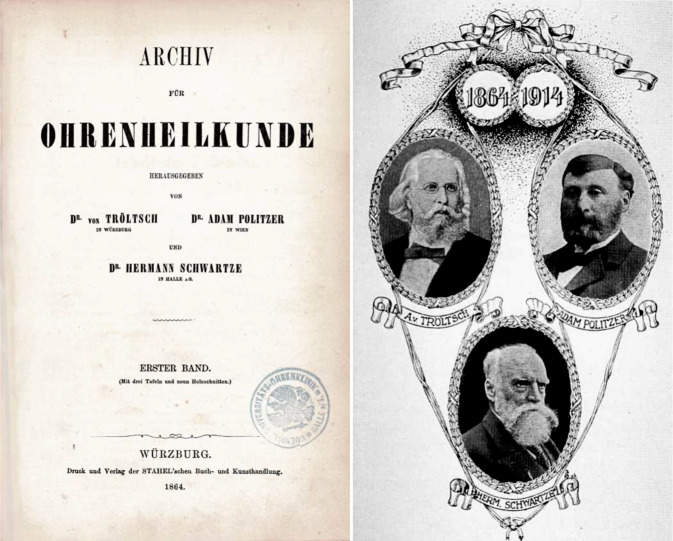

Im Jahr 1864 wurde mit dem *Archiv für Ohrenheilkunde *die erste wissenschaftliche Zeitschrift unseres Fachgebietes von Anton von Tröltsch (1829–1890) aus Würzburg, Adam Politzer (1835–1920) aus Wien und Hermann Schwartze (1837–1910) aus Halle (Saale) gegründet [31, 34]. Heute besteht die Zeitschrift als *European Archives of Oto-Rhino-Laryngology and Head & Neck* und deren ehemals als Beiheft erschienenen *HNO *weiter fort (Abb. 1 und 2). Auch die *Laryngo-Rhino-Otologie *hat alte Wurzeln: zunächst als *Monatsschrift für Ohrenheilkunde *bzw.* Monatsschrift für Ohrenheilkunde sowie für Kehlkopf‑, Nasen‑, Rachenkrankheiten* als Beilage der *Allgemeinen Medizinischen Zentralzeitung* und später dann als eigenständige Zeitschrift (Abb. 3).

**Figure 2:**
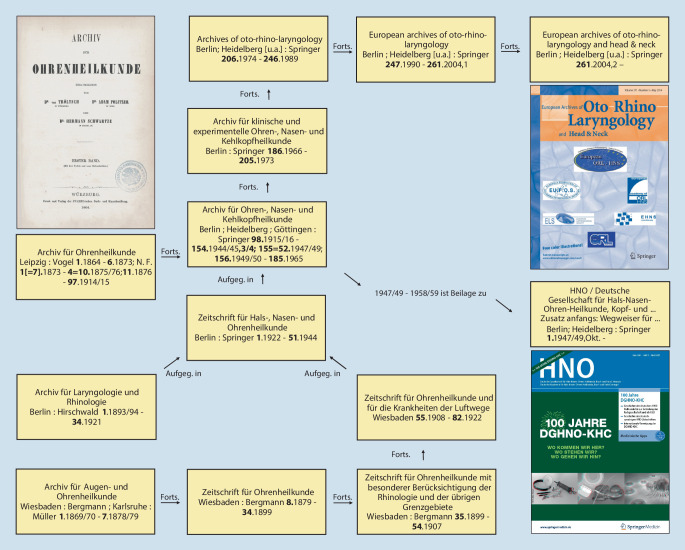

**Figure 3:**
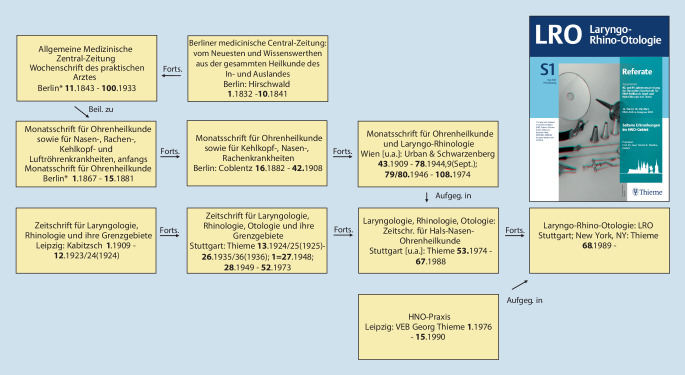

**Figure 4:**
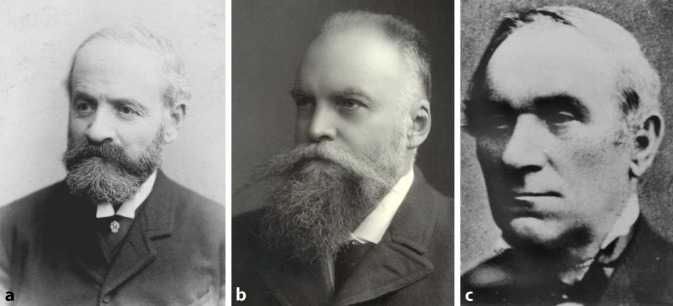

Vor circa 150 Jahren, in den 60er-Jahren des 19. Jahrhunderts, wurden mit Salomon Moos (1831–1895) in Heidelberg, Hermann Schwartze (1837–1910) in Halle (Saale) sowie Friedrich Voltolini (1819– 1889) in Breslau die ersten außerordentlichen Professoren in unserem Fachgebiet ernannt. Interessant ist, dass Moos sich zunächst für innere Medizin habilitiert hatte. Voltolini wurde 1868 außerordentlicher Professor für Otologie und Laryngologie und darf somit als erster akademischer Vertreter des Gesamtfachs Hals-Nasen-Ohren-Heilkunde in Deutschland bezeichnet werden (Abb. 4).

**Figure 5:**
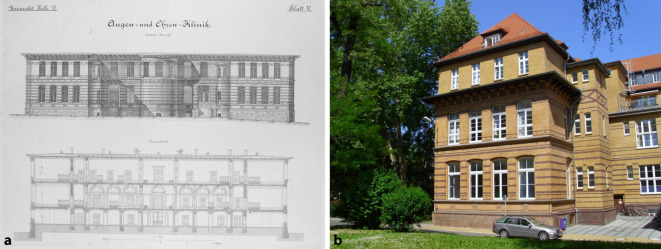

Im Jahre 1884 wurde in Halle (Saale) mit der *Königlichen Universitäts-Ohrenklinik* das erste Klinikgebäude in Deutschland speziell für die stationäre Behandlung von Erkrankungen in unserem Fachgebiet errichtet (Abb. 5).

**Figure 6:**
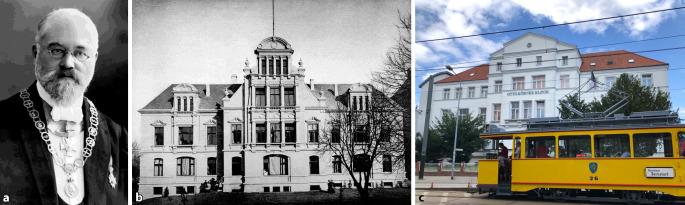

Im Jahre 1899 wurde in Rostock die erste Universitätsklinik für das Gesamtfach eröffnet, als deren Direktor Otto Körner im Jahre 1901 zum ersten ordentlichen Professor für das Gesamtfach Hals-Nasen-Ohren-Heilkunde in Deutschland ernannt wurde (Abb. 6).

Bis zur vollständigen Akademisierung und Etablierung unseres Fachgebietes an den Universitäten war es also ein langer Weg. Auch deshalb sollten wir den an einigen Universitäten beobachteten momentanen Praktiken, die HNO-Klinik-Leitungen mit außerplanmäßigen Professoren und Professorinnen, solchen in Teilzeit und Nebentätigkeit oder aber mit akademischen Leitern und Leiterinnen außerhalb des Fachgebietes Hals-Nasen-Ohren-Heilkunde, Kopf- und Hals-Chirurgie zu besetzen, sehr kritisch begegnen.

**Figure 7:**
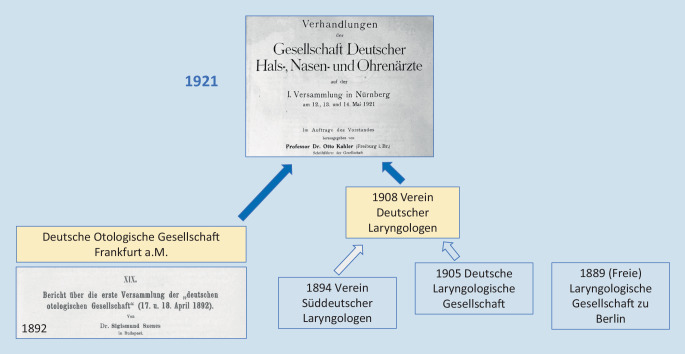

Der Mai 2021 zeichnet ein besonderes Datum für die *Deutsche Gesellschaft für Hals-Nasen-Ohren-Heilkunde, Kopf- und Hals-Chirurgie. *Genau 100 Jahre zuvor, bei ihrer 1. Jahresversammlung vom 12.–14. Mai 1921 in der Stadtbibliothek im „Wespennest“ in Nürnberg, entstand die Rechtsvorgängerin unserer wissenschaftlichen Fachgesellschaft, die *Gesellschaft deutscher Hals‑, Nasen- und Ohren-Ärzte*, durch den Zusammenschluss der *Deutschen Otologischen Gesellschaft* mit dem *Verein Deutscher Laryngologen *(Abb. 7).

Die Wachowskis lassen Morpheus in dem Film *Matrix* sagen: „Wie es scheint, kommt das Schicksal nicht ohne einen Sinn für Ironie“ („Fate, it seems, is not without a sense of irony.“). Und wenn man die COVID-19-Pandemie als Schicksalsschlag betrachtet, dann müssen wir feststellen, dass sich 100 Jahre nach dieser Vereinigung ein „Otologe im Herzen“ mit einem im weitesten Sinne „Laryngologen im Herzen“ zusammengetan haben und nun, 100 Jahre nach der Gründung unserer Fachgesellschaft, zum ersten Mal in unserer Geschichte als „Doppelspitze“ diesem – und nun kommt schon wieder ein Novum – ersten vollständig virtuellen oder Online-Kongress unserer Fachgesellschaft als gemeinsame Kongresspräsidenten vorstehen. Wir haben uns vorgenommen, dies nun alle 100 Jahre so zu tun und dem Präsidium einen entsprechenden Beschluss vorzulegen. ;-)

**Figure 8:**
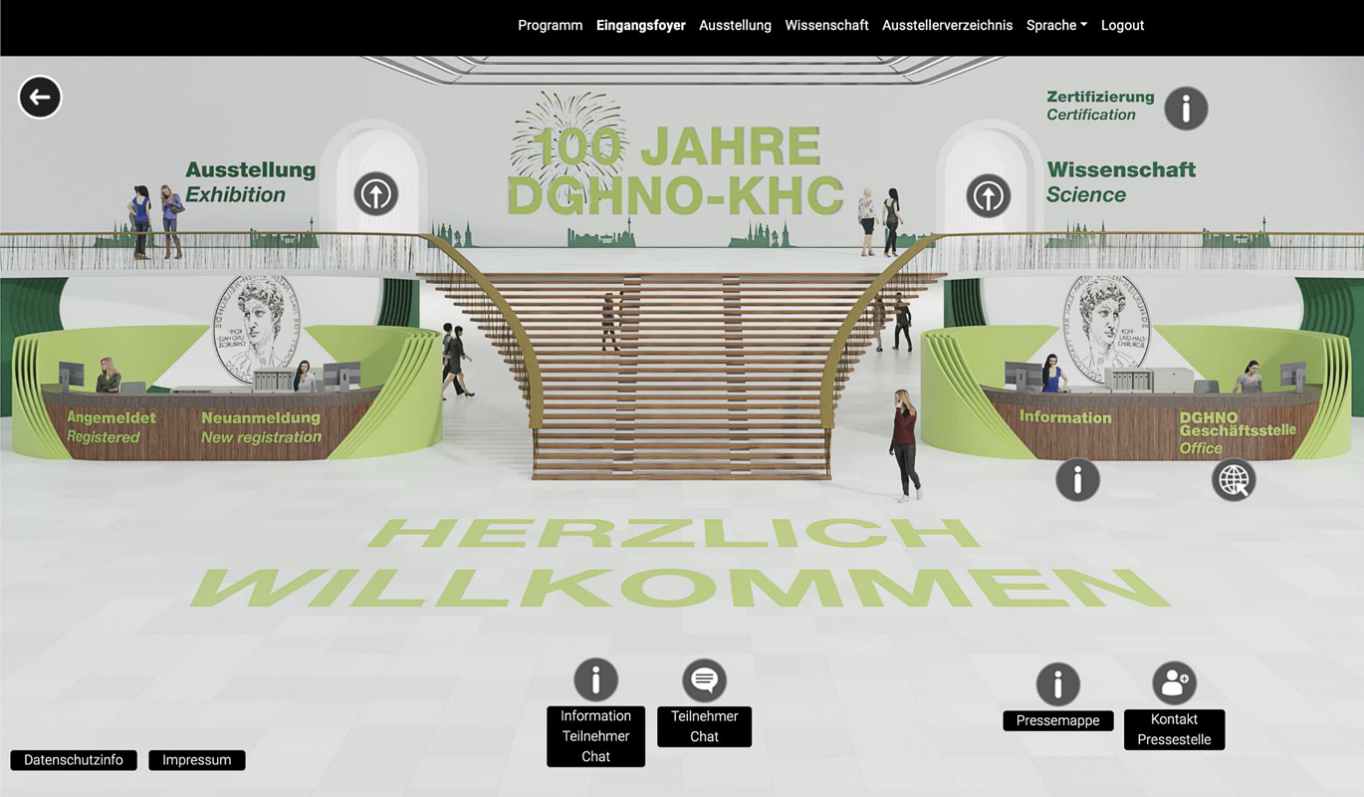

Lieber Andreas, ich möchte mich hier bei Dir ganz herzlich für die wirklich gute Zusammenarbeit in diesem doch so besonderen Jahr der COVID-19-Pandemie bedanken. (Abb. 8).

Auf der 20. Jahresversammlung 1949 in Karlsruhe wurde die *Gesellschaft Deutscher Hals‑, Nasen- und Ohrenärzte *umbenannt in *Deutsche Gesellschaft der Hals-Nasen-Ohren-Ärzte* und auf der 39. Jahresversammlung 1968 in Bad Reichenhall in *Deutsche Gesellschaft für Hals-Nasen-Ohren-Heilkunde, Kopf- und Hals-Chirurgie e.* *V., Bonn*.

Zur Frage „Wo kommen wir her?“ gehört aber auch die Auseinandersetzung mit der Rolle der HNO-Ärzte und HNO-Ärztinnen in der Zeit des Nationalsozialismus sowie während der DDR-Diktatur. Auch im HNO-Fachgebiet gab es exponierte Wissenschaftler und Wissenschaftlerinnen, die durch ihre Tätigkeit dem nationalsozialistischen Regime aktiv zugearbeitet haben. Als Beispiele seien die aktive Mitarbeit in den Erbgerichtshöfen und die wissenschaftlichen Arbeiten zu erblich bedingter Schwerhörigkeit und Gehörlosigkeit, die Leitfäden und Gutachten, auf Basis derer tausende Menschen zwangssterilisiert und Schwangere zu Abtreibungen gezwungen wurden, genannt. Damit waren HNO-Fachvertreter und -Fachvertreterinnen unmittelbar an der Umsetzung der NS-Rassenideologie beteiligt und trugen eine wesentliche Mitverantwortung an dem Leid, das den Betroffenen nach 1933 widerfuhr [41, 47, 48].

Bei den heutigen Preisverleihungen unserer wissenschaftlichen Fachgesellschaft heißt es meist „… soll in erster Linie die Persönlichkeit ehren und die Vorbildfunktion des Preisträgers hervorheben …“. 1993, auf der 64. Jahresversammlung unserer Fachgesellschaft in Münster, wurde dem ehemaligen Ordinarius am gleichen Orte, Karl Mündnich, die Ehre der Verdienstmedaille in Gold der DGHNO zuteil. Während der Zeit des Nationalsozialismus war Mündnich Obersturmbannführer der *Leibstandarte Adolf Hitler*, einer handverlesenen Truppe, der man sicher kein harmloses Mitläufertum bescheinigen darf [23, 24].

Auch hat mich bei meinen Recherchen irritiert, dass bei Berufungen in den 1950er-Jahren sowohl in der damaligen Bundesrepublik als auch in der damaligen DDR die Augen vor der NS-Vergangenheit offensichtlich in einigen Fällen fest zugedrückt worden sind. Woldemar Tonndorf z. B. gratulierte Oscar Wagener aus Göttingen in einem Nachruf noch nachträglich quasi zur „Juden-freien HNO-Klinik“ („… Wagener hatte von jeher mit sicherem Instinkt jeden Mitarbeiter abgelehnt, an dessen Rassenzugehörigkeit der leiseste Zweifel bestand – er hatte sich 1933 keine Vorwürfe zu machen …“ [44]). Im Jahr 1951 wurde Tonndorf als HNO-Ordinarius an die Karl-Marx-Universität in Leipzig berufen – nur wenige Jahre, bevor Ernst Bloch (Sie erinnern sich an den Anfang dieses Vortrages!) wegen der freien Rede die Lehrbefugnis in Leipzig verlor.

Dass in Diktaturen Nähe zur Staatsführung die Karriere fördert, ist sicher nicht verwunderlich; wir müssen aber auch an die denken, deren Entwicklung als Wissenschaftler und Kliniker in diesen Zeiten unterbunden wurde oder Schlimmeres.

**Figure 9:**
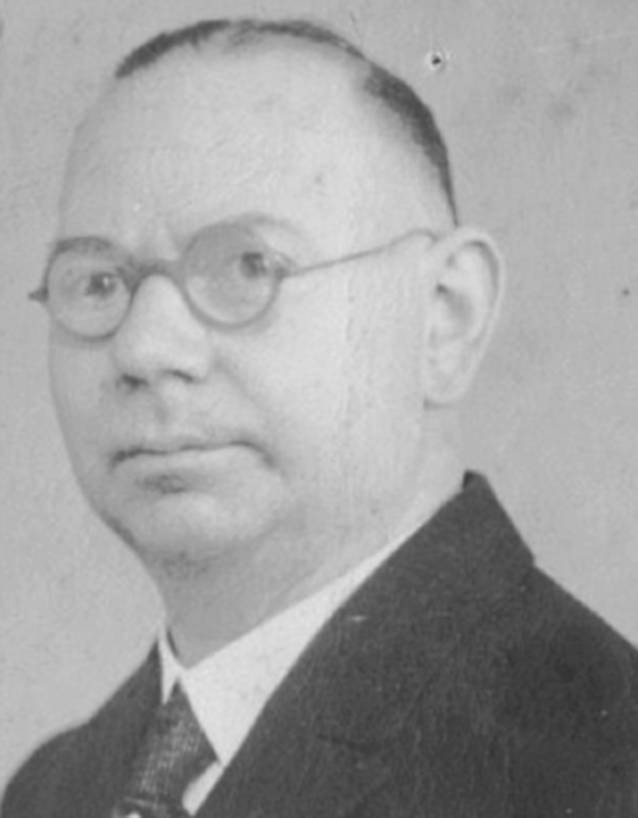

**Figure 10:**
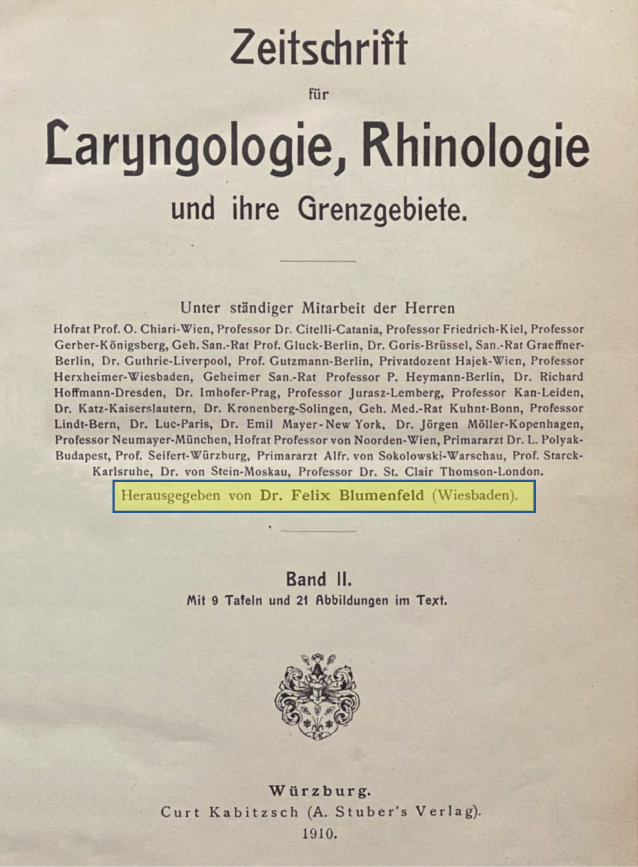

Als Beispiele genannt seien hier Josef Cohen (1873–1955), Chefarzt in Köln, der 1933 des Amtes enthoben wurde ([27]; Abb. 9), sowie Felix Blumenfeld (1864–1947) aus Wiesbaden, 1909 Gründungsherausgeber und dann langjähriger Herausgeber der *Zeitschrift für Laryngologie, Rhinologie und ihre Grenzgebiete* – der späteren *Laryngo-Rhino-Otologie* –, bis zum durch die Nationalsozialisten erzwungenen Entzug seiner Herausgeberschaft im Jahr 1934 ([39, 42]; Abb. 10).

Während zahlreiche Institutionen und Fachgesellschaften in Deutschland ihre Rolle während des Nationalsozialismus bereits thematisieren und aktiv aufarbeiten oder aufgearbeitet haben, besteht hier für unsere Fachgesellschaft noch Handlungsbedarf. Daher hat das Präsidium der DGHNO-KHC beschlossen, die Geschichte der Deutschen HNO-Heilkunde vor, während und nach der Zeit des Nationalsozialismus wissenschaftlich begleitet aufarbeiten zu lassen [36].

Mehr zur Geschichte finden Sie in der aktuellen Ausgabe der Zeitschrift *HNO* [33] mit Beiträgen zur deutschen HNO-Geschichte bis zur Gründung unserer Fachgesellschaft 1921 und weiter ab 1921 [28, 29], aber auch zur Geschichte der deutschsprachigen HNO-Zeitschriften [42] und zur internationalen Vernetzung unserer Fachgesellschaft [32]. Hinweisen möchte ich auch auf zahlreiche Publikationen von Harald Feldmann zur Geschichte der HNO-Heilkunde (z. B. [9–12]) und auf den Übersichtsbeitrag von Karl Heinz Vosteen zur „Entwicklung der Hals-Nasen-Ohrenheilkunde im 19. Jahrhundert“, der 1996 im Jubiläumsband zu den akademischen Lehrstätten und Lehrern in der HNO-Heilkunde [46] erschienen ist, sowie auf den publizierten Festvortrag von Konrad Fleischer anlässlich des 75. Jahrestages der Gründung der Gesellschaft im Jahre 1996 [13].

**Figure 11:**
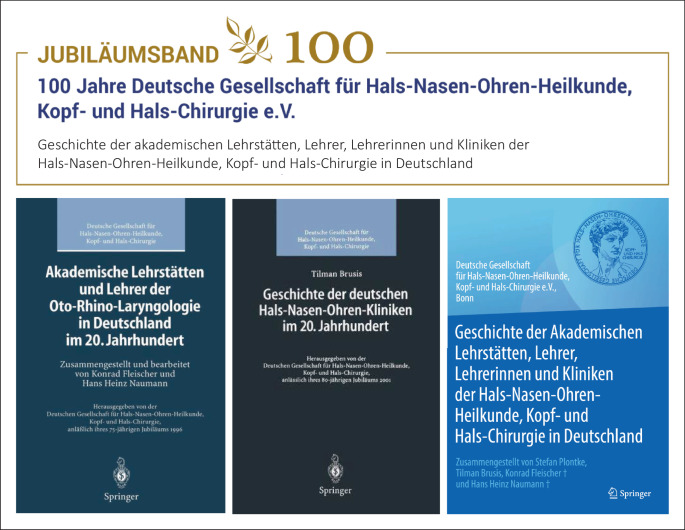

Eine Ergänzung wird das im Jubiläumsjahr unserer Fachgesellschaft geplante Buch *Geschichte der akademischen Lehrstätten, Lehrer und Lehrerinnen und Kliniken der Hals-Nasen-Ohren-Heilkunde, Kopf- und Hals-Chirurgie in Deutschland* sein. Es wird die beiden 1996 und 2001 erschienenen Vorgänger in einem Band vereinen und die Geschichte der Universitäts-HNO-Kliniken um die letzten 25 Jahre und die der nichtuniversitären HNO-Kliniken um die letzten 20 Jahre fortschreiben. Viele Entwicklungen der deutschen HNO-Heilkunde von ihren institutionellen Anfängen bis in das Jahr 2021 lassen sich auch aus den Kapiteln zu den jeweiligen Standorten ablesen ([1]; Abb. 11).

**Figure 12:**
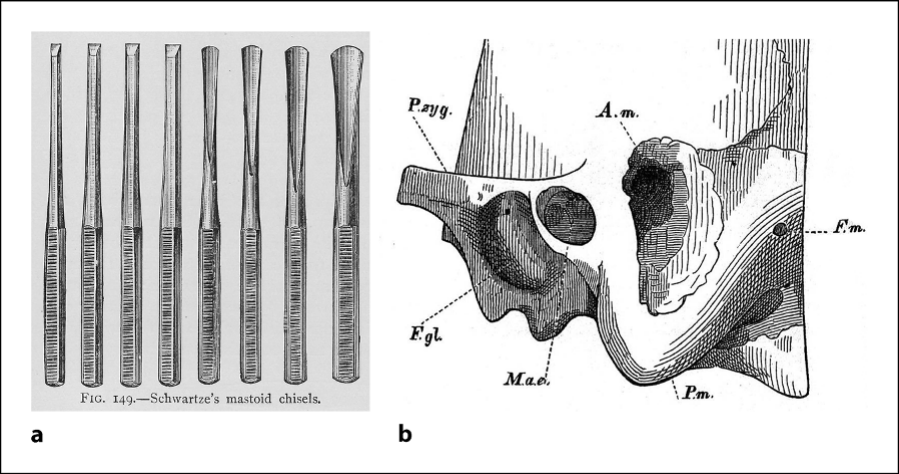

Ein wenig Lokalpatriotismus sei mir an dieser Stelle erlaubt. Daher möchte ich noch einmal zurückblicken auf den bei seiner Einführung sicher segensreichen Meißel in die Ohrchirurgie durch Hermann Schwartze (Abb. 12).

Dass dieser auch seine Risiken birgt, beschrieb Alfred Schulz van Treeck aus Berlin 1951 im *Lehrbuch für HNO-Heilkunde*: „Zur Vorsicht lässt man bei diesem Akt die vom Fazialisnerv innervierte Gesichtsmuskulatur durch einen Assistenten beobachten. Zuckungen verraten, dass der Fazialis in Gefahr ist, manchmal allerdings ist die beobachtete Zuckung die letzte, da sie bereits die Durchtrennung anzeigt“.

Das führt mich zum zweiten Teil meiner Präsidentenrede und der Frage:

## „Wo stehen wir?“

Hier sei zunächst eine kleine Auswahl genannt (Tab. 2).

**Table 2:** 

Die inzwischen etablierten Techniken der Tympanoplastik mit Titan-Mittelohrprothesen
Implantierbare elektronische Hörsysteme sowie Erfolge in der Innenohrchirurgie
Die Hörrehabilitation mit Cochleaimplantaten bei Gehörlosigkeit und partieller Taubheit
Die Defektrekonstruktion mit mikrovaskulär anastomosierten Transplantaten
Präzise intensitätsmodulierte Radiotherapie oder Strahlentherapie mit Schwerionen
Chemotherapie
Moderne Immuntherapien
Chirurgische Assistenzsysteme („Roboter“)
Intraoperative Bildgebung und Navigation
Bahnbrechende Fortschritte in der bildgebenden, histologischen und molekulargenetischen Diagnostik

Ganz im Zeichen der zweiten Frage „Wo stehen wir?“ steht unser Jubiläumskongress, der sich neben einigen historischen Aspekten mit den Vorträgen, Symposien, Rundtischgesprächen und „Oxford-Style-Debatten“ sowie der Einbindung der Industriepartner den wissenschaftlichen Hauptthemen „Qualität in der Medizin“ (als Thema der aufgrund der COVID-19-Pandemie ausgefallenen Jahresversammlung 2020) sowie „seltene Erkrankungen“ widmet [35].

Zur Beantwortung der Frage „Wo stehen wir?“ gehört aber auch der Blick über unsere eigenen Grenzen hinaus, und hier müssen wir feststellen, dass es weltweit erhebliche Unterschiede beim Zugang zur Gesundheitsversorgung gibt [3, 5, 21].

Zudem führt globale Fehlernährung derzeit zu mehr Krankheits- und Todesfällen als Gewalt, Konsum von Drogen, Alkohol und Tabak und ungeschützter Sex zusammen [49]. Von den weltweit 56 Mio. Todesfällen im Jahre 2012 waren 620.000 (ca. 1 %) durch Gewalt (davon 120.000 durch Krieg und 500.000 durch Kriminalität), 800.000 durch Selbstmord und ca. 1,5 Mio. durch Diabetes verursacht. Man könnte auch zusammenfassen: „Zucker ist heute gefährlicher als Schießpulver“ (zitiert aus: [17]).

Dies leitet über zum dritten Teil meiner Rede und damit zur dritten Frage:

## „Wo gehen wir hin?“

Stephen Hawking (1942–2018) fasste in seinem posthum erschienenen Buch *Kurze Antworten auf große Fragen* [18] die wirklich großen Fragen, denen wir uns stellen sollten, wie folgt zusammen (Tab. 3).

**Table 3:** 

1.	Gibt es einen Gott?
2.	Wie hat alles angefangen?
3.	Gibt es anderes intelligentes Leben im Universum?
4.	Können wir die Zukunft vorhersagen?
5.	Was befindet sich in einem schwarzen Loch?
6.	Sind Zeitreisen möglich?
7.	Werden wir auf der Erde überleben?
8.	Sollten wir den Weltraum besiedeln?
9.	Wird uns künstliche Intelligenz überflügeln?
10.	Wie gestalten wir unsere Zukunft?

Aus: Stephen Hawking, Kurze Antworten auf große Fragen (2018), [18]

Als wissenschaftliche Fachgesellschaft werden wir uns wohl nur mit sehr wenigen dieser Fragen beschäftigen.

Konkret müssen wir uns jedoch in den kommenden Jahren Herausforderungen in den folgenden Gebieten stellen (Tab. 4):

**Table 4:** 

1.	Spezialisierung versus Einheit des Fachgebietes
2.	Digitalisierung, „Big Data“ und künstliche Intelligenz
3.	Ökonomisierung/Kommerzialisierung
4.	Priorisierung und Ressourcenallokation (verbunden mit den ethischen Fragen, die sich hieraus ergeben und die z. B. beim Thema *seltene Erkrankungen*, aber auch im Rahmen der aktuellen COVID-19-Pandemie besonders deutlich werden)
5.	Ambulantisierung
6.	Bürokratisierung
7.	Feminisierung (in Bezug auf den Anteil beim ärztlichen Personal) und zunehmendes Interesse an Arbeit in Teilzeit
8.	Akademisierung/Entakademisierung
9.	Industrialisierung und Ökonomisierung der Forschung
10.	Personalisierung der Medizin
11.	Technisierung – chirurgische Assistenzsysteme/Robotik
12.	Paradigmenwechsel: steigender Anteil an medikamentösen Therapien und Gentherapie
13.	Aus- und Weiterbildung

Wir bekennen uns auch 100 Jahre nach der Gründung unserer Fachgesellschaft zur **Einheit des Fachgebietes Hals-Nasen-Ohren-Heilkunde, Kopf- und Hals-Chirurgie**. Dennoch ergibt sich aus der Breite des Faches, der Vielzahl von (seltenen) Erkrankungen, dem rapiden medizinisch-technischen Fortschritt, den ökonomischen Rahmenbedingungen unter gleichzeitigem Qualitätsanspruch bei Diagnostik und Therapie sowie aufgrund der bei einem chirurgischen Fachgebiet inhärent erforderlichen und auf operativer Erfahrung und „Übung“ basierenden chirurgischen Expertise die **Notwendigkeit der Spezialisierung** auch innerhalb unseres Fachgebietes. Die zukünftigen medizinischen Versorgungsstrukturen müssen der Sicherstellung einer ambulanten und stationären HNO-fachärztlichen Grundversorgung in der Breite, aber auch einer spezialisierten, technisierten, interdisziplinären und interprofessionellen Versorgung an Zentren gerecht werden. Diese Spezialisierung ist auch erforderlich, um die Grenzgebiete unseres Faches durch größtmögliche Kenntnisse, Fähigkeiten und Fertigkeiten gegenüber „Begehrlichkeiten“ anderer Fachgebiete absichern zu können, während sie gleichwohl der interdisziplinären Zusammenarbeit zum Nutzen für die betroffenen Patienten und Patientinnen dient.

Auch die ***Weiterbildung*** in unserem Fachgebiet muss den hohen Anforderungen des einerseits sehr breiten und andererseits sehr spezialisierten Wissens in unserem Fachgebiet gerecht werden. Sie darf nicht minimalisiert werden und muss auf soliden Finanzierungsmodellen basieren – im stationären wie auch im ambulanten Bereich. Eine weitgehende oder gar vollständige Weiterbildung im Fach Hals-Nasen-Ohren-Heilkunde in rein ambulanten Versorgungsstrukturen halte ich für einen falschen Weg. Erfahrungen aus der Augenheilkunde zeigen uns bereits die Folgen dieser Fehlentwicklungen. Wenn die Weiterbildung aus den großen Kliniken in die Praxen ausgelagert wird, dann schmälert das den Erfahrungswert dieser Generation(en) von Weiterbildungsassistentinnen und -assistenten. Und das kann nicht gut sein für die Patientinnen und Patienten, die unserer Hilfe bedürfen – und nicht zuletzt sind ja auch wir selbst später diese Patientinnen und Patienten.

Mit der notwendigen Spezialisierung eng verbunden ist auch die ***präklinische und klinische Forschung***. Dabei ist es wichtig, aktuelle Probleme in der Forschung und Forschungsförderung zu adressieren. Ein hoher Anteil der biomedizinischen Studien weist methodische Mängel auf. Dazu gehören am häufigsten: (a) ein schlechtes Versuchsdesign („underpowered“), eine unsaubere Statistik, die selektive Wiedergabe von Daten und das einseitige Publizieren von positiven Ergebnissen. Oft ist das Studiendesign gar nicht geeignet, eine bestimmte Fragestellung zu beantworten. John Ioannidis aus Stanford kam 2005 sogar zu dem Schluss: „Most published research findings are false“, und zu viele Ergebnisse sind nicht reproduzierbar [20]. Wissenschaft und Wissenschaftsförderung haben sich zu sehr als Markt etabliert, wobei die Bewertung des wissenschaftlichen Erfolges problematisch ist. Als Fehlanreiz hat sich Quantität (Fördermittel und Publikationszahlen) statt Qualität und Kreativität entwickelt.

Die Förderung von wenigen Universitäten im Rahmen der Exzellenzinitiative der Bundesregierung „… hat mit einem klassischen Organisationsproblem zu kämpfen. Die Anreizstruktur der Initiative wird häufig so interpretiert, dass sie vor allem die Quantität des wissenschaftlichen Outputs prämiert. Die Qualität bleibt auf der Strecke. … Die Zahl der Publikationen pro Professur ist an allen deutschen Universitäten gestiegen, an Nicht-Exzellenzuniversitäten sogar stärker als an Exzellenzuniversitäten. Doch im gleichen Zeitraum sank die durchschnittliche Zahl der Zitationen pro Veröffentlichung – und zwar besonders stark in der Gruppe der Exzellenzuniversitäten …“ [19].

Auch die Frage nach der Bedeutung der Größe von Universitäten und Forschergruppen wird kontrovers diskutiert und ist differenziert zu betrachten.

Eine Forschergruppe aus Chicago und Evanston hat 65 Mio. Artikel, Patente und Softwareprodukte eines Zeitraums von 60 Jahren ausgewertet. Sie kamen zu einem interessanten Ergebnis: Große Teams tendieren zur Weiterentwicklung existierender Ideen, kleine Teams tendieren zu „disruptiven“ Technologien und Wissenschaft. Die Gruppe schussfolgerte daraus, dass sowohl kleine also auch große Teams essenziell für eine blühende Wissenschaft und Technologie sind. Wissenschaftspolitik muss daher eine Diversität von Forschungsgruppen fördern [50]. Dies ist insbesondere von Bedeutung, da „… die Deutsche Forschungsgemeinschaft systematisch die größeren Einrichtungen bevorzugt“. Durch Oligarchisierung in der Universitätslandschaft wird aber das wissenschaftliche Potenzial nicht ausgeschöpft [15]. Ebenso wie einzelne Kliniken können auch Forschungsgruppen und Forschungsverbünde ab einer bestimmten Größe ihre „Resonanzfrequenz“ verlieren.

Mit dem ***Deutschen Studienzentrum HNO*** wurde ein wichtiger Schritt hin zur Stärkung der evidenzbasierten Medizin in der HNO und zur klinischen Studienkultur in unserem Fachgebiet getan. Unter anderem der ökonomische Druck behindert jedoch an den Universitäten die Förderung des wissenschaftlich-medizinischen Nachwuchses. Wir müssen hier als Fachgesellschaft aktiv einer Entakademisierung entgegenwirken, den „Clinician Scientist“ fördern und der Entwicklung in Richtung einer Aus- und Weiterbildung nach dem Modell der „Arztschule“ entgegenwirken. Spezialisierung bedeutet eher mehr Akademisierung in der HNO.

Strukturell wird uns das Thema ***Ambulantisierung*** als wichtige Veränderung in den nächsten Jahren begleiten. Wir werden hier darauf achten müssen, dass technisch aufwendige und komplexe Operationen unter ambulanten Bedingungen zu den gleichen Qualitätsstandards wie aktuell im stationären Setting durchgeführt und auch adäquat vergütet werden [6]. Eine Vereinbarkeit dieser Entwicklung mit den aktuell geltenden Finanzierungssystemen einschließlich der Fallpauschalen im stationären Bereich ist nicht gegeben. Es ist auch noch unklar oder sogar fraglich, ob ein höherer Anteil ambulanter HNO-Operationen die Antwort auf und die Lösung für den Pflegemangel in Deutschland sein wird. Die Gleichung „ambulant = niedrigerer Aufwand = billig“ ist sicher falsch. Auf jeden Fall wird die sektorenübergreifende medizinische Versorgung in der Behandlung nicht nur komplexer Erkrankungen im HNO-Bereich zunehmend an Bedeutung gewinnen.

Beim Thema ***Digitalisierung und künstliche Intelligenz*** soll vor dem Hintergrund der Geschichte auch erinnert werden an Alan Turing (1912–1954), der einen großen Teil der theoretischen Grundlagen für die moderne Informations- und Computertechnologie schuf, und an Konrad Zuse (1919–1995), den Konstrukteur des ersten funktionsfähigen Computers der Welt.

„Leben und Gesundheit der Menschen in Deutschland könnten besser geschützt werden, wenn die Möglichkeiten der Digitalisierung im Gesundheitswesen verantwortlich und wissenschaftlich sinnvoll genützt würden.“ Zu diesem Schluss kommt der *Sachverständigenrat Gesundheit* in seinem aktuellen Gutachten, das im März 2021 Herrn Minister Spahn übergeben wurde (zitiert nach Pressemitteilung zu [14]). „Ziel soll die Neuausrichtung der Gesundheitsversorgung sein: hin auf ein digitales, ein systematisch lernendes Gesundheitssystem“. Dabei sollen Gesundheitsdaten nicht in die falschen Hände fallen. Zugleich müssen sie in die richtigen Hände gelangen können. Der Sachverständigenrat empfiehlt hier eine Strategie zur Digitalisierung des Gesundheitswesens. Diese beinhaltet z. B. (i) Rahmenbedingungen der Digitalisierung einschließlich Abwägungen im Bereich Datenschutz, (ii) Empfehlungen zur elektronischen Patientenakte und (iii) Empfehlungen zur Datennutzung für gezieltere Forschung, Prävention, Diagnostik und Therapie (nach [14]).

Im Bereich künstliche Intelligenz und maschinelles Lernen gibt es auch im HNO-Fachgebiet Anstrengungen und Aktivitäten. Eine Übersichtsarbeit zum Thema identifizierte 90 Studien, die den Einsatz künstlicher Intelligenz insbesondere in den Bereichen Bildanalyse, Sprachanalyse, Gen-Analyse sowie Elektroencephalogramm, Elektrokardiogramm und Apnoe-Hypopnoe-Index anhand klinischer Daten untersuchten und berichtete erste Erfolge, postulierte aber auch weiteren Forschungsbedarf [43].

Digitalisierung, künstliche Intelligenz und Big Data werden als Konzept angepriesen, mit dem wir an der Schwelle zu einer Präzisionsmedizin stehen, die in Kürze fehlerfrei diagnostiziert und therapiert. Mit der richtigen technischen Aufrüstung für die Beherrschung der ungeheuren Datenmengen warten am Horizont schon das Ende des Zufalls und damit eine fehlerfreie Medizin. So die Versprechungen. Genaueres Hinschauen beschert allerdings Ernüchterung. Von den üblichen drei Dimensionen der Technologiebewertung – nämlich Nutzen, Risiko und Kosten – werden die letzten beiden tunlichst ignoriert und die Bewertung auf ein – meist völlig überhöhtes – Nutzenversprechen reduziert. Vor allem aber ist wichtig, dass selbst bei größter Technikgläubigkeit die Auswirkung auf die Menschlichkeit in der Medizin nicht übersehen werden darf. (Zitiert aus: [2]).

Unsere Anstrengungen – unbedingt auch auf politischer Ebene – müssen sich auch gegen die Verschwendung von Ressourcen durch ***Bürokratisierung***, eine unangemessene ***Ökonomisierung (bzw. Kommerzialisierung)*** und die Ausweitung von ***Fehlanreizen*** richten.

Dabei ist eine enge Zusammenarbeit – trotz in manchen Aspekten und Sachfragen unterschiedlichen Überzeugungen oder zum Teil differierender Interessenlagen – mit den Ärzten und Ärztinnen anderer Fachgebiete und innerhalb unseres Fachgebietes zwischen wissenschaftlicher Fachgesellschaft und Berufsverband BV-HNO unbedingt erforderlich.

Ökonomisches Handeln ist auch im Gesundheitswesen wichtig. Wir aber müssen entscheiden: Wollen wir ein **Gesundheitswesen oder einen Gesundheitsmarkt**? Ist Krankenversorgung Daseinsfürsorge oder Regulation durch den Markt? Wenn wir denken, der Markt regle das schon, dann müssen wir feststellen, dass der Markt vor allem sich selbst dient! Der Staat aber hat Verantwortung für Leben und Gesundheit seiner Bürgerinnen und Bürger. Staatlich garantierte Einnahmen aus einem solidarisch finanzierten System sollten keine Quelle für lukrative Renditeobjekte sein. Wir müssen auf dem HNO-Gebiet eine flächendeckende Daseinsfürsorge für die Bevölkerung auf hohem Niveau auch durch Realisierung verschiedener Versorgungsniveaus schaffen, anstatt Patientinnen und Patienten durch „Reklame“, „Portalpraxen“ oder Ähnliches mit dem Zweck der Gewinnmaximierung zu lenken. Auch müssen wir unsere eigene Rolle als „Angestellte“ im System hinterfragen: Sind wir „freie Heilberufler“ oder einfache „Erfüllungsgehilfen“ für die Generierung von Profiten für Aktiengesellschaften, ausländische Rentenfonds oder andere finanzstarke Fremdinvestoren [16, 26, 30, 37, 45]?

**Figure 13:**
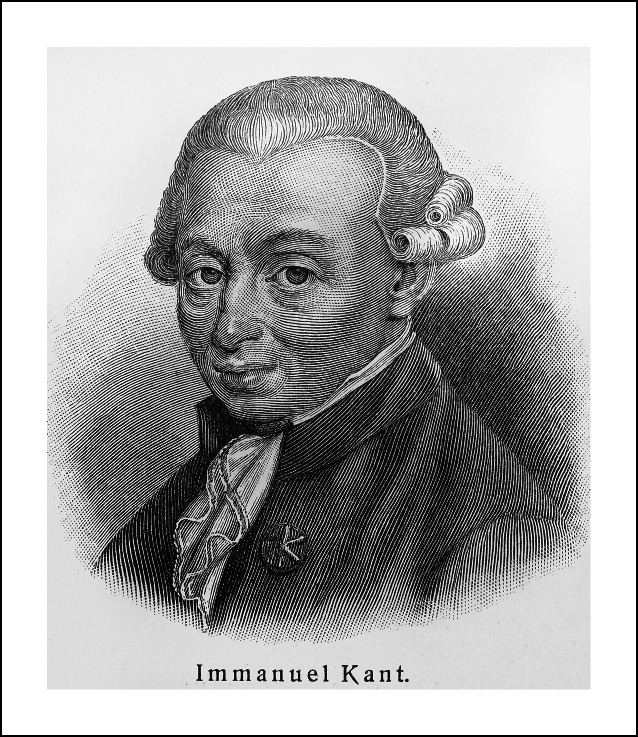

Auf die Frage **„Ist der Patient Mittel zum Zweck oder Zweck selbst?“ **gibt wiederum der Blick in die Geschichte eine klare Antwort: In der Kant’schen „Selbstzweckformel“ ist das Instrumentalisierungsverbot anderer Menschen und damit von Patientinnen und Patienten verankert: „Handle so, dass du die Menschheit sowohl in deiner Person, als in der Person eines jeden anderen jederzeit zugleich als Zweck, niemals bloß als Mittel brauchst“ ([22]; Abb. 13).

Wir müssen die ärztliche Verantwortung für den Versorgungsprozess in weiten Strecken erhalten bzw. zurückgewinnen, damit am Ende – ganz im Kant’schen Sinne – der Mensch immer Zweck seines Handelns bleibt.

Vielen Dank für Ihr Ohr!

Stefan K. Plontke

Präsident 2020/21

der Deutschen Gesellschaft für Hals-Nasen-Ohren-Heilkunde, Kopf- und Hals-Chirurgie

## References

[1] Anonymous (in press) Geschichte der akademischen Lehrstätten, Lehrer und Lehrerinnen und Kliniken der Hals-Nasen-Ohren-Heilkunde, Kopf- und Hals-Chirurgie in Deutschland. Spinger, Heidelberg

[2] Antes G, Thielscher C (2019). Der Arzt behält die Deutungshoheit trotz KI. Dtsch Arztebl.

[3] Biesinger E, Dillinger J, Groth A (2019). Ear aid in Myanmar. HNO.

[4] Bloch E (1959). Das Prinzip Hoffnung.

[5] Dazert S, Heinrich D (2019). Helping people to help themselves: 10 years of successful graduate medical training in otorhinolaryngology in Rwanda (Africa). HNO.

[6] Deitmer T, Dietz A, Delank KW, Plontke SK, Welkoborsky HJ, Dazert S (2021) Ambulantes Operieren in der HNO-Heilkunde in Deutschland [Outpatient Surgery in German ENT]. Laryngorhinootologie. 10.1055/a-1418-9745. Epub ahead of print 10.1055/a-1418-9745 33822330

[7] Dench EB (1896). Diseases of the ear.

[8] Dolgner AD (1996). Die Bauten der Universität Halle im 19. Jahrhundert: Ein Beitrag zur deutschen Universitätsbaugeschichte.

[9] Feldmann H (2004). 200 years testing hearing disorders with speech, 50 years german speech audiometry—a review. Laryngorhinootologie.

[10] Feldmann H (2016). Bilder aus der Geschichte der Hals-Nasen-Ohren-Heilkunde.

[11] Feldmann H (2002). Historical evolution of cochlear implants—from Volta to multichannel intracochlear stimulation. Laryngorhinootologie.

[12] Feldmann H (2000). Oscar Wilde. Medical observations on the 100th anniversary of his death 30 November 2000. Laryngorhinootologie.

[13] Fleischer K (1996). 75 Jahre Deutsche Gesellschaft für Hals-Nasen-Ohrenheilkunde, Kopf- und Halschirurgie; Festvortrag anläßlich des 75. Jahrestages der Gründung der Gesellschaft. DGHNO-KHC, Homepage der Deutschen Gesellschaft für Hals-Nasen-Ohrenheilkunde, Kopf- und Halschirurgie.

[14] Gerlach F, Greiner W, Jochimsen B, Von Knalle C, Meyer G, Schreyögg J, Thürmann P (2021). Digitalisierung für Gesundheit – Ziele und Rahmenbedingungen eines dynamisch lernenden Gesundheitssystems, Gutachten 2021.

[15] Grözinger G (2021). Groß ist gut, klein bloß charmant? Hochschulgröße als Erfolgsfaktor. Forsch Lehre.

[16] Haentjes C, Huppertz C, Dippmann I, Kloppmann S (2021). Helios-Kliniken: Millionengewinne und knappes Personal.

[17] Harari YN (2017). Homo Deus: Eine Geschichte von Morgen.

[18] Hawkin S (2018). Kurze Antworten auf grosse Fragen.

[19] Holzer B (2020) Mehr Quantität statt Qualität: Die Salami-Taktik der exzellenten Universitäten. Frankf Allg Ztg: https://www.faz.net/aktuell/wissen/forschung-politik/mehr-quantitaet-statt-qualitaet-dysfunktionalitaeten-der-exzellenzinitiative-16938040.html. Zugegriffen: 28.02.2022

[20] Ioannidis JP (2005). Why most published research findings are false. PLoS Med.

[21] Jungehulsing M, Markmiller U, Schroder U (2019). MEDCARE for People in Eritrea: a report on 9 years’ help through self-help. HNO.

[22] Kant I (1781) Grundlegung zur Metaphysik der Sitten. Gesammelte Schriften Akademieausgabe. De Gruyter, , S 429 (Nachdr. d. Ausg. 1903)

[23] Klee E (2015). Auschwitz, die NS-Medizin und ihre Opfer.

[24] Klee E (2016). Das Personenlexikon zum Dritten Reich. Wer war was vor und nach 1945.

[25] Ladenthin V (2021). Nice to have, but useless? Warum wir die historischen Fächer brauchen. Forsch Lehre.

[26] Maio G (2009). Should modern medicine become a service industry? An ethical appraisal of a market-oriented medicine. Ther Umsch.

[27] Maune S, Brusis T (in press) Krankenhaus Köln-Holweide der Kliniken der Stadt Köln. In: DGHNO-KHC (Hrsg) Geschichte der akademischen Lehrstätten, Lehrer und Lehrerinnen und Kliniken der Hals-Nasen-Ohren-Heilkunde, Kopf- und Hals-Chirurgie in Deutschland. Springer, Heidelberg

[28] Mudry A (2021). Otorhinolaryngology as “Made in Germany” since 1921: an international perspective. HNO.

[29] Mudry A, Mlynski R, Kramp B (2021). History of otorhinolaryngology in Germany before 1921. HNO.

[30] N.N. (2021) SPD Sachsen-Anhalt und Verdi warnen vor Klinikübernahme durch Ameos. aerztblatt.de. https://www.aerzteblatt.de/nachrichten/108767/SPD-Sachsen-Anhalt-und-Verdi-warnen-vor-Klinikuebernahme-durch-Ameos. Zugegriffen: 28.02.2022

[31] Peinhardt J, Plontke SK, Mudry A (2015). The Archiv fur Ohrenheilkunde (Archive of Otology): a structural analysis of the first 50 years (1864–1914). Eur Arch Otorhinolaryngol.

[32] Pirsig W (2021). On the international networking of the German Society of Otorhinolaryngology, Head and Neck Surgery. HNO.

[33] Plontke SK (2021). 100 years of the German Society of Otorhinolaryngology, Head and Neck Surgery: Where do we come from? Where are we? Where are we going?. HNO.

[34] Plontke SK (2015). Otology Jubilee: 150 years of the Archiv fur Ohrenheilkunde “Where do we come from?—Where are we?—Where are we going?”. Eur Arch Otorhinolaryngol.

[35] Plontke SK (2021). Seltene Erkrankungen und Hals-Nasen-Ohren-Heilkunde, Kopf und Halschirurgie. Laryngorhinootologie.

[36] Plontke SK, Stöver T (2021). Präsidiumssitzung 15. und 16. März 2021. HNO Inf.

[37] Pohl M, Sieber U, Wandt L (2021). Streit um Kinderklinik: Schwere Vorwürfe gegen Asklepios-Konzern.

[38] Santayana G (1905). The life of reason: the phases of human progress.

[39] Schriftleitung und Verlag (1948). Professor Felix Blumenfeld. Z Laryngol Rhinol Otol.

[40] Schwartze H, Eysell A (1873). Über die Eröffnung des Warzenfortsatzes, zum Operationsverfahren. Arch Ohrenheilkd.

[41] Schwarz M (1935). „Ererbte Taubheit“: Grundzüge zur Erkennung ererbter Hörstörungen soweit sie das Gesetz zur Verhütung erbkranken Nachwuchses betreffen.

[42] Stasche N, Bärmann M (2021). History of the German-language ENT journals. German version. HNO.

[43] Tama BA, Kim DH, Kim G (2020). Recent advances in the application of artificial intelligence in otorhinolaryngology – head and neck surgery. Clin Exp Otorhinolaryngol.

[44] Tonndorf W (1942). Oskar Wagener zum Gedächtnis. Arch Ohren Nasen Kehlkopfheilkd.

[45] Von Weizsacker F, Maio G (2010). Ethical insolvency?. Dtsch Med Wochenschr.

[46] Vosteen KH, DGHNO-KHC (1996). Als Einführung: Die Entwicklung der Hals-Nasen-Ohrenheilkunde im 19. Jahrhundert. Akademische Lehrstätten und Lehrer der Oto-Rhino-Laryngologie in Deutschland im 20. Jahrhundert.

[47] Wade B (2021). Arbeitskreis Universität Tübingen im Nationalsozialismus: Stellungnahme zur NS-Vergangenheit von Prof. Dr. med. Max Schwarz.

[48] Weichbold V, Zorowka P (2008). Zwangssterilisation bei erblicher Taubheit im Dritten Reich: Auswirkung auf die wissenschaftliche Diskussion innerhalb der HNO-Ärzteschaft [Forced sterilisation of the hereditary deaf in the Third Reich: Its reflection in the scientific ENT literature]. HNO.

[49] Willett W, Rockstrom J, Loken B (2019). Food in the Anthropocene: the EAT-Lancet Commission on healthy diets from sustainable food systems. Lancet.

[50] Wu L, Wang D, Evans JA (2019). Large teams develop and small teams disrupt science and technology. Nature.

[51] https://commons.wikimedia.org/wiki/File:Rudolf_Voltolini.jpg. Zugegriffen: 10. Jan. 2022

[52] Deutscher Verein für Öffentliche Gesundheitspflege (1901) Festschrift der XXVI. Versammlung des Deutschen Vereins für Öffentliche Gesundheitspflege gewidmet von der Stadt Rostock. Raths- und Universitäts-Buchdruckerei von Adler’s Erben, Rostock. http://purl.uni-rostock.de/rosdok/ppn879171189. Zugegriffen: 24. Jan. 2022

